# Proofreading of Peptide—MHC Complexes through Dynamic Multivalent Interactions

**DOI:** 10.3389/fimmu.2017.00065

**Published:** 2017-02-08

**Authors:** Christoph Thomas, Robert Tampé

**Affiliations:** ^1^Institute of Biochemistry, Biocenter, Goethe University Frankfurt, Frankfurt am Main, Germany

**Keywords:** adaptive immunity, antigen presentation, MHC, peptide-loading complex, peptide editing, quality control, tapasin, molecular tug-of-war

## Abstract

The adaptive immune system is able to detect and destroy cells that are malignantly transformed or infected by intracellular pathogens. Specific immune responses against these cells are elicited by antigenic peptides that are presented on major histocompatibility complex class I (MHC I) molecules and recognized by cytotoxic T lymphocytes at the cell surface. Since these MHC I-presented peptides are generated in the cytosol by proteasomal protein degradation, they can be metaphorically described as a window providing immune cells with insights into the state of the cellular proteome. A crucial element of MHC I antigen presentation is the peptide-loading complex (PLC), a multisubunit machinery, which contains as key constituents the transporter associated with antigen processing (TAP) and the MHC I-specific chaperone tapasin (Tsn). While TAP recognizes and shuttles the cytosolic antigenic peptides into the endoplasmic reticulum (ER), Tsn samples peptides in the ER for their ability to form stable complexes with MHC I, a process called peptide proofreading or peptide editing. Through its selection of peptides that improve MHC I stability, Tsn contributes to the hierarchy of immunodominant peptide epitopes. Despite the fact that it concerns a key event in adaptive immunity, insights into the catalytic mechanism of peptide proofreading carried out by Tsn have only lately been gained *via* biochemical, biophysical, and structural studies. Furthermore, a Tsn homolog called TAP-binding protein-related (TAPBPR) has only recently been demonstrated to function as a second MHC I-specific chaperone and peptide proofreader. Although TAPBPR is PLC-independent and has a distinct allomorph specificity, it is likely to share a common catalytic mechanism with Tsn. This review focuses on the current knowledge of the multivalent protein–protein interactions and the concomitant dynamic molecular processes underlying peptide-proofreading catalysis. We do not only derive a model that highlights the common mechanistic principles shared by the MHC I editors Tsn and TAPBPR, and the MHC II editor HLA-DM, but also illustrate the distinct quality control strategies employed by these chaperones to sample epitopes. Unraveling the mechanistic underpinnings of catalyzed peptide proofreading will be crucial for a thorough understanding of many aspects of immune recognition, from infection control and tumor immunity to autoimmune diseases and transplant rejection.

## Introduction

Presentation of antigenic peptides on major histocompatibility complex class I (MHC I) molecules is fundamental to the recognition of infected and cancerous cells by the immune system (1). Peptides derived from intracellular pathogens or endogenous self-antigens by proteasomal degradation and peptidase trimming are transported from the cytosol into the endoplasmic reticulum (ER) lumen by the ATP-binding cassette (ABC) transporter associated with antigen processing (TAP), a component of the peptide-loading complex (PLC) that resides in the ER membrane (2–4). In the ER, the peptides are then processed by specific proteases and loaded onto MHC I molecules, which subsequently travel to the cell surface where they are scanned by CD8^+^ T-lymphocytes. However, the peptides are not indiscriminately loaded onto MHC I molecules, but rather selected for high affinity and stability in an optimization step called peptide proofreading or editing (5). While TAP already achieves some degree of selectivity, the actual proofreading step, ensuring that stable peptide–MHC I complexes are presented to the immune system (6), is catalyzed by two MHC I-specific chaperones called tapasin (Tsn) (7–9) and TAP-binding protein-related (TAPBPR) (10). The selection of high-affinity peptide epitopes is essential: it gives T cells enough time to scan the peptide–MHC I complexes and prevents the exchange of endogenous peptides against exogenous peptides on the MHC I molecules, which would distort the presentation of the intracellular proteome status (1).

This review will describe the major players in catalyzed peptide proofreading, in particular Tsn, with a focus on the molecular mechanisms of the proofreading activity, the associated protein plasticity, and the dynamics of the interaction partners. Key information about these aspects of antigen presentation has recently been obtained mainly by structural, biochemical, and computational studies.

## Molecular Environment and Architecture of Tsn

**Figure 1:**
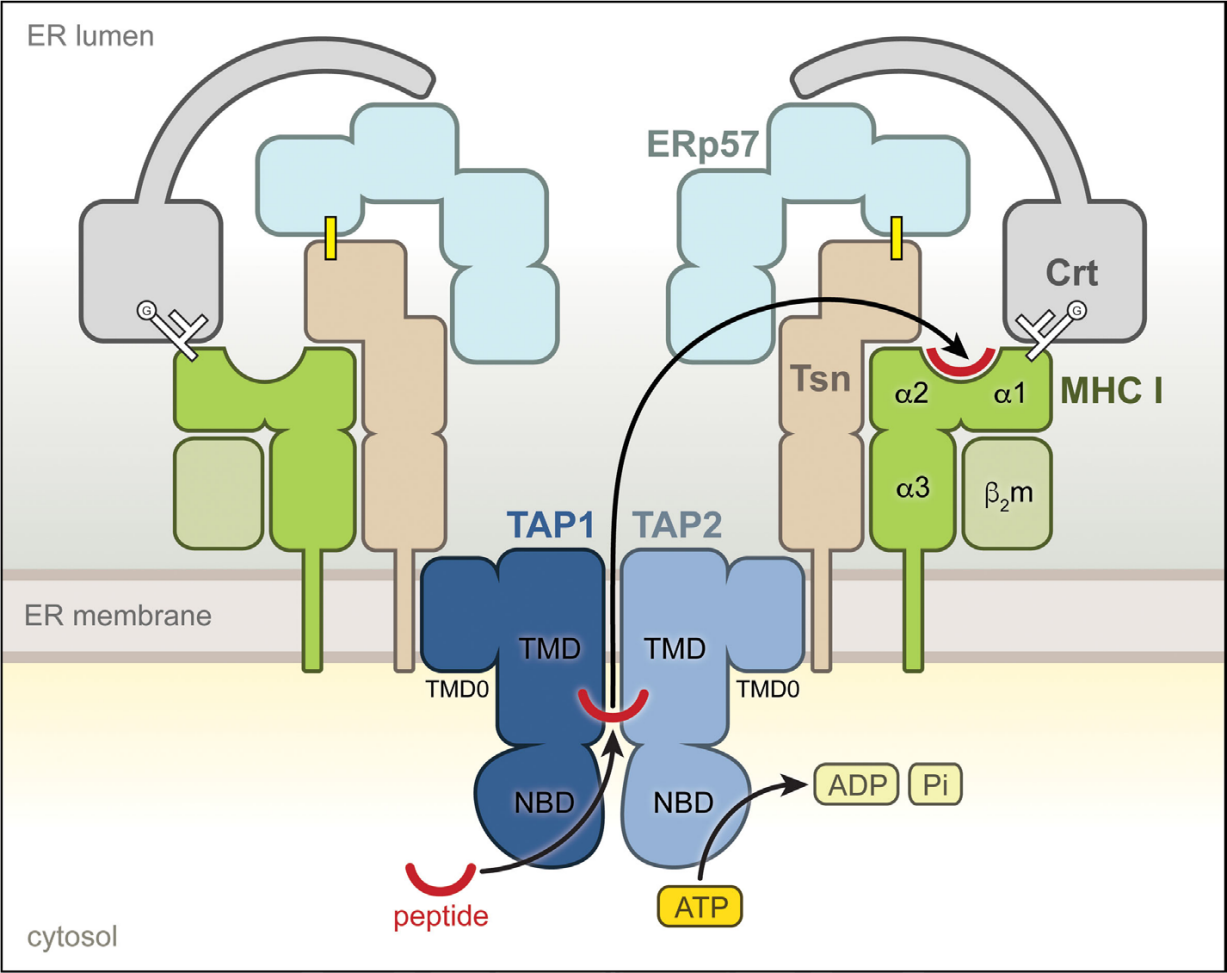
**Molecular environment of tapasin (Tsn) within the peptide-loading complex (PLC)**. Structural organization of the PLC. The individual components of the PLC are shown according to their domain organization. Tsn, covalently linked to the oxidoreductase ERp57 through a disulfide bridge (yellow line), interacts *via* its transmembrane region with the heterodimeric ATP-binding cassette transporter TAP1/2, which shuttles antigenic peptides across the endoplasmic reticulum (ER) membrane in an ATP-dependent manner. The monoglucosylated (G) *N*-glycan of the MHC I is shown as white branched lines. This multivalent interaction network localizes recruited calreticulin-associated MHC I molecules directly at the peptide source, facilitating selection of high-affinity epitopes.

Tapasin is a type I transmembrane protein of the ER with a short cytoplasmic tail and an ER-lumenal region of ~400 amino acids, which harbors the catalytic activity (11). Together with the ABC transporter TAP, the lectin-like chaperone calreticulin (Crt), and the disulfide isomerase ERp57 (ER protein 57), it forms the PLC (12) (Figure 1). Tsn is conjugated with ERp57 *via* a disulfide bond (7, 13), which is important for its full activity (12), and primarily interacts with TAP through its transmembrane domain, thereby forming a bridge between peptide-receptive MHC I and the peptide translocator TAP (14–17). Apart from its catalytic activity, the Tsn–ERp57 conjugate therefore plays a crucial architectural role and ensures the stability of the PLC (13, 18). The structure of Tsn–ERp57 has been solved by X-ray crystallography (19), revealing that the lumenal part of Tsn is L-shaped and consists of two domains, a distal N-terminal fusion domain of a seven-stranded β barrel and an immunoglobulin (Ig)-like fold followed by a C-terminal (membrane-proximal) IgC1 domain. The covalently linked ERp57 adopts the conformation of a twisted U, and its main function appears to reside in a facilitated recruitment of properly glycosylated MHC I molecules (12, 18–20), which are bound through their glycan moiety by Crt that also associates with ERp57 *via* its P domain (21–23) (Figure 1).

## Tsn Differentially Interacts with Optimally and Suboptimally Loaded MHC I

Initial hints that Tsn is a vital component of antigen processing came from experiments with a Tsn-deficient cell line (24–26) and Tsn-knockout mice (27, 28). Loss of Tsn leads to a drastic reduction in MHC I surface expression (16, 24, 27, 28). Using the ER-lumenal domains of Tsn and the HLA-B*08:01 heavy chain, zippered by Jun and Fos leucine peptides, it could be shown that Tsn increases the dissociation of certain peptides from MHC I (29), but the degree of Tsn sensitivity of different peptides could not be directly correlated with their intrinsic dissociation half-lifes. In the same study, experiments with peptides lacking either the N- or C-terminus indicated that catalysis by Tsn involves disruption of conserved hydrogen bonds at the C-terminal end of the peptide-binding groove. The authors concluded that Tsn selects high-affinity peptides by generating an energy barrier through widening of the MHC I peptide-binding groove. Wearsch and Cresswell used soluble, recombinantly generated Tsn–ERp57 conjugate and cell extracts of Tsn-negative cells transfected with HLA-B8 to provide evidence that Tsn–ERp57 promotes the exchange of intermediate- and low-affinity peptides for high-affinity epitopes (12). These initial demonstrations of Tsn-catalyzed peptide dissociation and discrimination of unstable peptide–MHC I complexes have later been confirmed by analyzing peptide loading onto the mouse MHC I allele H2-K^b^ using isolated microsomes (30). Thus, Tsn is more than a simple facilitator as it had initially been postulated in a study that was denying Tsn any ability to discriminate between low- and high-affinity peptides (31). It is remarkable that Tsn is able to enrich MHC I molecules with high-affinity epitopes despite an estimated 1,000-fold excess of low-affinity over high-affinity peptides in its environment (32). Because MHC I molecules are intrinsically flexible and unstable without tightly bound peptides (33–37), Tsn also acts as a chaperone during peptide exchange, stabilizing peptide-free MHC I (29). Several attempts have been made to mechanistically explain the observed effects of Tsn on MHC I (5, 12, 29, 30, 38, 39), and the principles of its activities are now clearer, thanks to several recent studies, including the description of the Tsn–ERp57 crystal structure (19). To further biochemically dissect the Tsn–MHC I interaction and the peptide-editing process *in vitro*, a tethering approach was employed that incorporated ERp57 and used recombinant biotinylated Tsn and MHC together with dimeric neutravidin to mimic the structural organization of Tsn–ERp57 and MHC I within the PLC. By combining this strategy with photo-cleavable peptide to synchronize and follow the catalytic process in real time, it was possible to demonstrate that Tsn increases the dissociation rate of low- and intermediate-affinity (suboptimal) peptide epitopes up to 10-fold (40). The exchange of suboptimal peptides for high-affinity ones turned out to be extremely slow in the absence of Tsn. Furthermore, the experiments provided unequivocal evidence that Tsn is able to discriminate between optimally and suboptimally loaded MHC I (40). This differential interaction of Tsn with MHC I, depending on the peptide cargo, is key to its ability to help selecting immunodominant peptide epitopes.

## Mechanistic Model of Tsn-Catalyzed Peptide Proofreading: A Molecular Tug-of-War

Differential antibody binding experiments and mutational analyses based on the Tsn–ERp57 structure helped to narrow down the main site of interaction with MHC I to a conserved region in the N-terminal domain of Tsn including residues E185, R187, Q189, and Q261 (19). MHC I residues that influence the interaction with Tsn (41–46) lie in two lumenal regions of the heavy chain, primarily on the same side as the α_2-1_ and α_2-2_ helices that form the flanking wall on one side of the peptide-binding groove. The crystal structure of a peptide-deficient non-classical MHC I (47) and MD simulations with HLA-A*02:01 (48) demonstrated that the binding groove in the peptide-free state adopts a more open conformation than in the peptide-bound state and is characterized by increased flexibility in the α_2-1_ helix close to the F pocket. A certain degree of flexibility of α_2-1_ had already been proposed earlier, based on structural comparisons (49–51). In contrast to the plasticity of the α_2-1_ helix region, the A-pocket region close to the binding region of the peptide N-terminus is significantly more rigid (48, 52–54). Since Tsn chaperones the peptide-free conformation, it has been speculated to do so by stabilizing the α_2-1_ helix in a position that leaves the binding groove in an open, peptide-receptive state (19).

**Figure 2:**
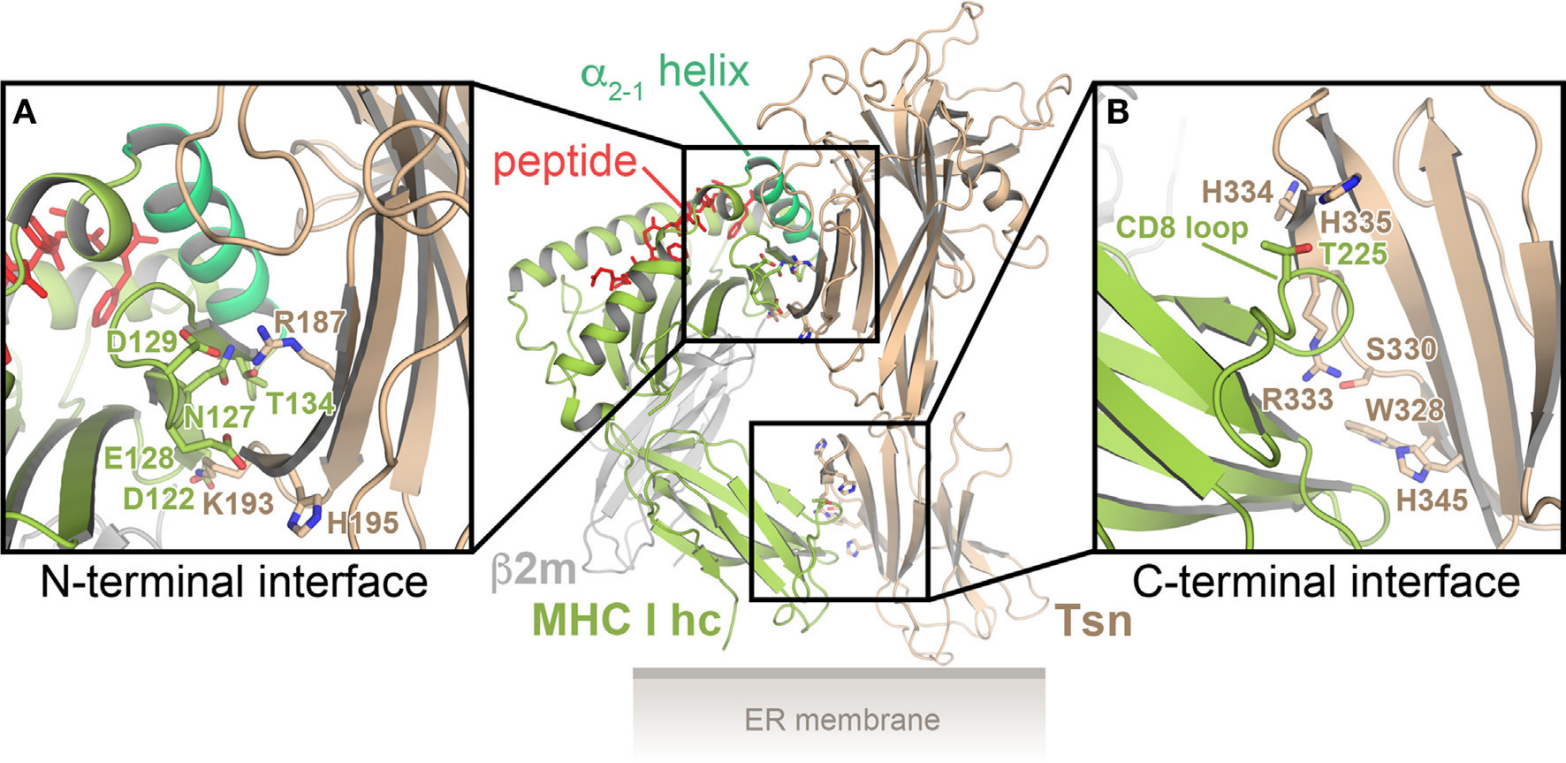
**Model of the tapasin (Tsn)–major histocompatibility complex class I (MHC I) interaction**. Cartoon representation of the predicted Tsn–MHC I complex, based on multi-microsecond all-atom MD simulations, for which the crystal structures of the lumenal portions of Tsn and HLA-B*44:02 were used as starting models (40, 55). There are two distinct interfaces, one between the N-terminal (distal) domain of Tsn and the α_2-1_-helix region of the MHC I heavy chain (MHC I hc) **(A)**, the other between the membrane-proximal domain of Tsn and the α_3_ domain of the MHC I hc **(B)**. Residues predicted to be part of the interfaces are shown as sticks in the close-up views. β2m, β2-microglobulin.

One possible interface for binding of the α_2-1_ helix is the conserved patch of residues in the N-terminal domain of Tsn. This notion of Tsn–MHC I interaction is supported by recent multi-microsecond all-atom MD simulations of the Tsn–MHC I complex in the peptide-bound and peptide-free state, for which the crystal structures of the lumenal portions of Tsn and HLA-B*44:02 were used as starting points (40, 55): two distinct interfaces were observed, one between the Tsn N-terminal domain and the MHC α_2_ domain, the other between the C-terminal domain of Tsn and the α3 domain of the MHC heavy chain (Figure 2). In the N-terminal interface, Tsn contacts the MHC α_2-1_ helix, the α_2-1/2_ hinge, and the underside of the β-sheet floor (β strands #7 and #8). The α_2-1_ helix is embraced and stabilized by Tsn in a conformation that maintains the peptide-binding groove in an open state. The MD simulations predict that the C-terminal interface consists of the CD8-binding site of the MHC and a cluster of basic Tsn residues (40, 55). The predicted Tsn contact residues are consistent with previous studies of potential Tsn interface residues (56–60). To establish the C-terminal interface, the membrane-proximal domain of Tsn has to rotate with respect to its position in the X-ray structure. This is made possible by a flexible linker connecting the two domains of Tsn, which acts like a hinge that gives Tsn a substantial degree of plasticity and allows the domains to move relatively to each other (55). Intriguingly, stable interactions in the N-terminal interface are more numerous in the peptide-free state than in the peptide-bound state, and, consequently, the F pocket of the peptide-binding groove is widened by 1–2 Å in the absence of peptide, most likely leading to a reduction in peptide affinity.

**Figure 3:**
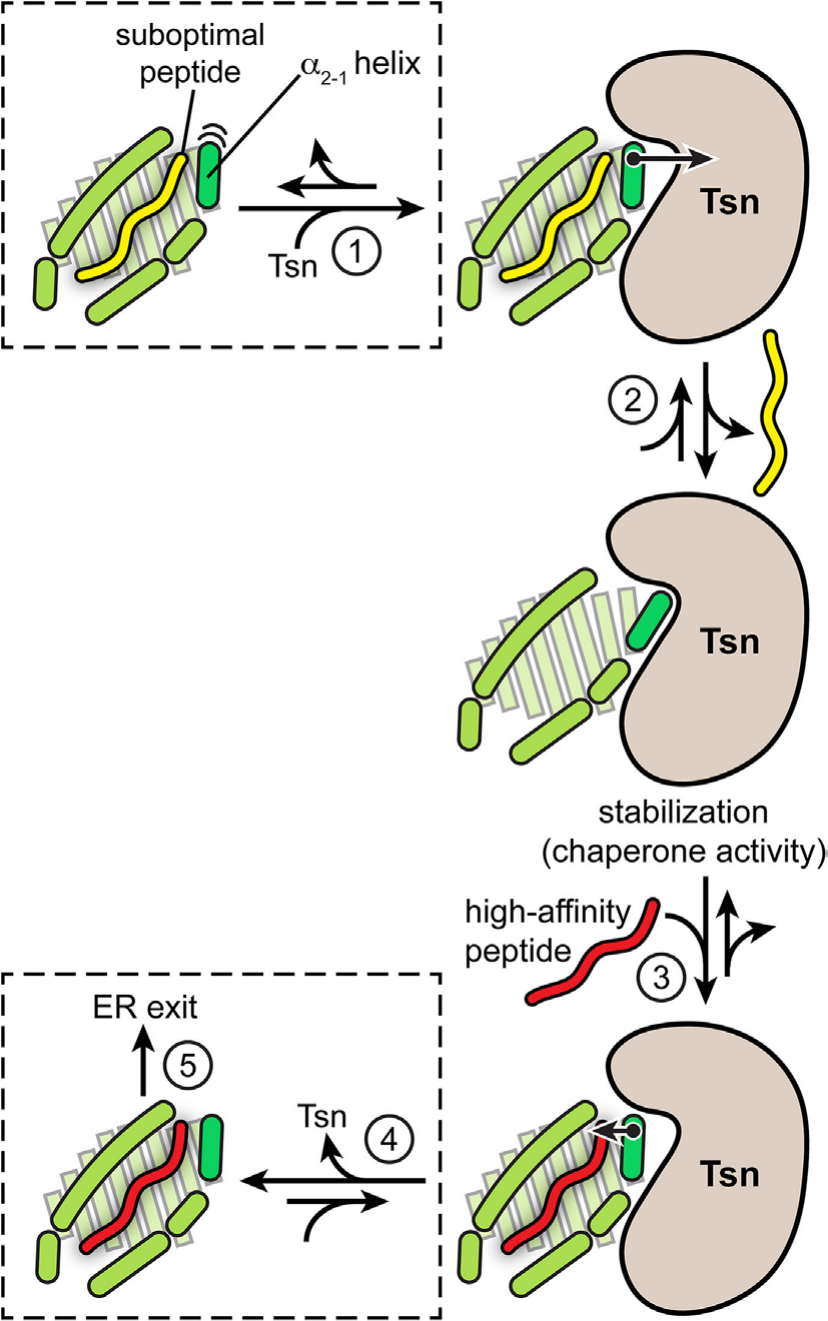
**Proposed model of tapasin (Tsn)-catalyzed peptide proofreading**. According to the model of Tsn-catalyzed peptide proofreading, Tsn scans the quality of major histocompatibility complex class I (MHC I)-bound peptides with regard to their affinity by sensing and acting on the α_2-1_ helix, a structural element close to the C-terminal anchor region of the peptide (F pocket). Intrinsic flexibility of the α_2-1_ helix is depicted by cartoon-blur semicircles. Peptide dissociation in the absence of Tsn can result in partial unfolding of the MHC molecule. Upon being confronted with a suboptimally loaded MHC I molecule (step 1), Tsn presumably stabilizes an open conformation of the binding groove by interacting with the α_2-1_ helix, inducing peptide dissociation and stabilizing the resulting empty MHC (step 2). Only high-affinity peptides can subsequently compete with Tsn over the α_2-1_ helix to tighten the binding groove again (step 3). This lowers the Tsn–MHC affinity and eventually triggers Tsn dissociation (step 4). As a result, the peptide repertoire presented on MHC I at the cell surface is enriched with high-affinity peptide epitopes capable of triggering an immune response (step 5). The quintessence of the MHC I peptide-proofreading mechanism might be considered as a tug-of-war between Tsn and the peptide over the α_2-1_ helix and the opening/closing of the binding groove.

**Figure 4:**
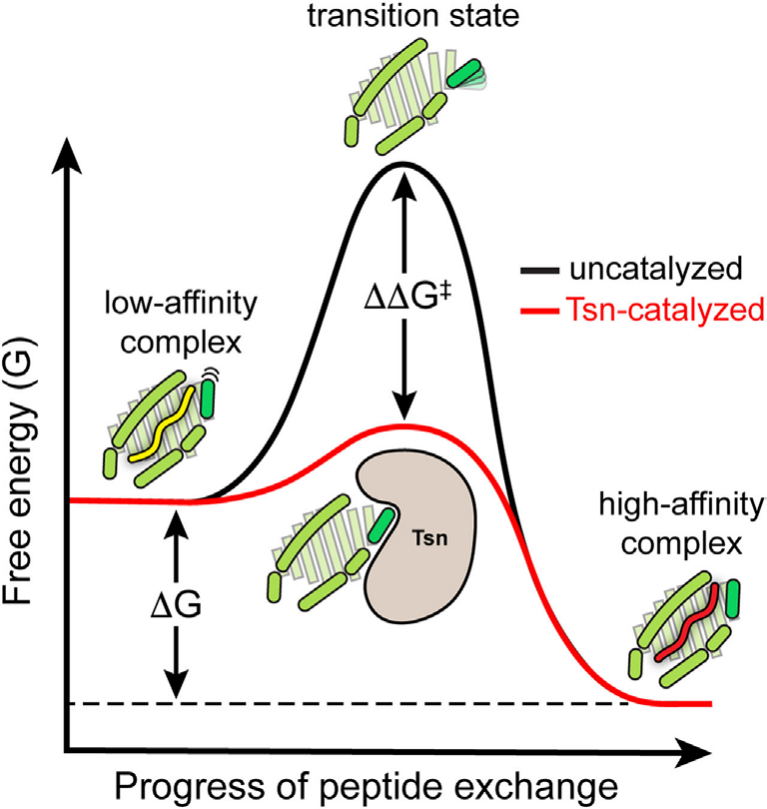
**Energy level diagram of the peptide exchange reaction**. Energy level diagram of the un-catalyzed and catalyzed peptide exchange reaction (energy levels are qualitative and not drawn to scale). In its function as a peptide exchange catalyst, tapasin stabilizes the high-energy intermediate of the empty MHC I molecule. Stabilizing this high-energy intermediate lowers the energy barrier (ΔΔG^‡^) of the exchange reaction and hence increases the rate of peptide exchange toward the thermodynamically most favored high-affinity peptide.

In summary, the catalytic working cycle of Tsn and its putative mechanism of action can be described as follows (Figure 3): Tsn monitors the quality of MHC I-associated peptides in terms of affinity by probing and acting on the α_2-1_ helix, a specific structural element of the peptide-binding groove close to the C-terminal anchor region of the peptide. Upon encountering a suboptimally loaded MHC I molecule, Tsn shifts the conformational equilibrium to an open conformation of the binding groove by interacting with the α_2-1_ helix, thus inducing peptide dissociation and stabilizing the resulting peptide-free MHC. Only subsequent binding of a high-affinity peptide can compete with Tsn over the α_2-1_ helix to close the binding groove again. This lowers the affinity of MHC I for Tsn and finally triggers Tsn dissociation (40). The result is a peptide repertoire displayed on MHC I that is enriched with high-affinity peptide epitopes, which are able to release Tsn from MHC I (29, 40). In this proposed mechanism of peptide editing, Tsn and the peptide might be considered as two opponents in a tug-of-war over the α_2-1_ helix and the opening/closing of the binding groove. In its function as a peptide exchange catalyst, Tsn stabilizes a transition state, namely the high-energy intermediate of the peptide-free MHC I (Figure 4). Stabilizing this high-energy intermediate lowers the energy barrier of the peptide exchange reaction and consequently increases its rate. The stabilization of the peptide-free MHC I state relative to the peptide-bound state has been determined to be in the range of −8 kJ/mol (40). The resulting accelerated exchange kinetics allows sampling of the peptidome for high-affinity epitopes. Tsn essentially converts the un-catalyzed kinetically controlled peptide loading into a thermodynamically controlled process, facilitating the selection of high-affinity peptides from a pool mainly consisting of suboptimal epitopes (40). This notion of peptide-proofreading catalysis has been confirmed by kinetics simulation studies (61). The proposed mechanism is further corroborated by experimental results and MD simulations that ascribe a dominant role in determining the stability of peptide-bound MHC I to the F pocket region of the binding groove and the C-terminus of the peptide (35, 62–64).

The described model of catalyzed peptide editing also provides an explanation for the observation that different MHC I allomorphs vary in their dependence on Tsn (38, 40, 65, 66). The most striking examples of differential Tsn dependence are the two allomorphs HLA-B*44:02 and HLA-B*44:05. They only differ by a single residue at position 116 on the rigid floor of the peptide-binding groove, but exhibit a markedly different Tsn dependence: HLA-B*44:02 contains an aspartate and is strongly Tsn-dependent, whereas HLA-B*44:05 has a tyrosine and is Tsn-independent. While the crystal structures of the two allomorphs in the peptide-bound state are very similar (60, 67), MD simulations predict that upon dissociation of the peptide C-terminus, structural changes, mainly occurring in the mobile α_2-1_ helix region that contacts residue 116, are significantly more pronounced in HLA-B*44:02 than in HLA-B*44:05. The conformation of HLA-B*44:02 shifts toward an open F pocket, whereas HLA-B*44:05 eventually adopts a closed conformation similar to the peptide-bound state (68, 69). The tyrosine of HLA-B*44:05 appears to stabilize the α_2-1_ helix and thereby creates an overall more stable MHC I, which has lower affinity for Tsn. A more recent study, combining *in vivo* experiments with computational system models and MD simulations, came to the conclusion that a conformational intermediate of MHC I is central in the process of peptide selection, that the intrinsic ability of MHC I molecules to select high-affinity peptides correlates with protein plasticity, and that Tsn modulates the peptide-selector function by modifying MHC I plasticity *via* an allosteric coupling of the peptide-binding regions and the α_3_ domain of MHC I (65, 70). Besides additional *in silico* evidence, *in vitro* experimental data support the idea that plasticity is indeed an intrinsic property of MHC I proteins (37, 71–75). The prediction that a region of the α_3_ domain (residues 220-227) can communicate with the peptide-binding groove might indicate that the predicted C-terminal interface of the Tsn–MHC I complex plays an active role during catalysis. A correlation between conformational flexibility and Tsn dependence similar to the HLA-B*44:02/B*44:05 pair has been described for the two allomorphs HLA-B*27:05 and HLA-B*27:09 (76), the former one being associated with the inflammatory disease ankylosing spondylitis.

In conclusion, the α_2-1_ helix appears to be the most malleable part of the peptide-binding region, and this plasticity emerges as a central determinant in the ability of MHC I molecules to scan a diverse range of different peptides; the α_2-1_ helix is used by Tsn as a control element to improve the selector function of MHC I allomorphs.

## TAPBPR, a Tsn-Related Player in the MHC I Antigen Presentation Pathway

In the year 2000, a gene highly conserved among vertebrates and encoding a Tsn homolog was discovered (77) and named TAPBPR (78). Human TAPBPR shares 21% sequence identity with human Tsn and exhibits the same lumenal domain architecture. The structural homology between TAPBPR and Tsn has been confirmed by small-angle X-ray scattering (79). Just like Tsn, TAPBPR is IFNγ-inducible (80–82), recognizes peptide-receptive MHC I in the ER, and catalyzes peptide proofreading (10, 79, 83), thereby altering the peptide repertoire presented on MHC I at the cell surface (83). The inducibility by IFNγ suggests a function in the control of viral infection. Interestingly, a correlation between the expression level of TAPBPR and glioblastoma patient survival has been demonstrated, arguing for a role of TAPBPR in the immune recognition of tumors (84). TAPBPR was also found in the leukocyte nuclear envelope proteome, and even a role of TAPBPR in positioning chromosomes in the nucleus has been postulated (85). In this context, TAPBPR has been speculated to facilitate loading of MHC I with pioneer translation products (10). Based on mutational data and the fact that the binding of TAPBPR and Tsn to MHC I is mutually exclusive, it has been concluded that the MHC I interaction interfaces, and hence the mode of MHC I stabilization, are conserved between Tsn and TAPBPR (79, 86). Despite these common features, TAPBPR contrasts with Tsn in several ways: TAPBPR lacks the positively charged residue in the center of the transmembrane region and, therefore, does not interact with TAP (14), i.e., it is not an integral constituent of the PLC. MHC I antigenic peptide selection is thus not restricted to the PLC. Moreover, TAPBPR is independent of ERp57 and other ER chaperones (81), in spite of an unpaired cysteine in its lumenal portion. Furthermore, as TAPBPR has no ER retention motif, it is not restricted to the ER, but also found beyond the medial *Golgi* compartment (81). Finally, TAPBPR has a different MHC I and peptide specificity. TAPBPR does not bind a pseudo-empty HLA-A*01.01 (79) using the strategy of photo-cleavable peptides (40). But, like Tsn, TAPBPR catalyzes peptide exchange on HLA-A*02:01, albeit with a different peptide preference (83); the fact that both exchange catalysts are active toward HLA-A*02:01 could be one reason why HLA-A*02:01 surface expression is relatively unaffected as long as one of the two chaperones is present and is only diminished once both are absent (43, 83). TAPBPR also interacts with and is active toward HLA-B allomorphs, but weaker than for HLA-A*02:01 (83). Consequently, TAPBPR deficiency in IFNγ-induced cells changes the peptide repertoire presented by HLA-B allomorphs, and TAPBPR depletion severely impacts HLA-B*07:02 surface expression, even in the presence of Tsn (83). TAPBPR interaction is not restricted to human allomorphs, but can as well be observed in the context of the murine MHC I molecules H2-D^d^, H2-L^d^, and H2-D^b^, indicating that TAPBPR uses conserved structural features in MHC I for recognition (79).

Although it is now clear that TAPBPR is a *bona fide* peptide-editing catalyst, its exact cellular role during antigen presentation is still unclear. TAPBPR might be part of an antigen presentation pathway that runs in parallel to the classical PLC-dependent one (10). However, the currently available data seem to support the view of a distribution of responsibilities between Tsn and TAPBPR, i.e., that TAPBPR is an additional quality control checkpoint in the classical secretory antigen presentation pathway (83). Increased association between TAPBPR and MHC I has been seen in Tsn-deficient cells, implying that the two proteins work hand in hand (86). Initial peptide loading is probably carried out by Tsn within the PLC, and TAPBPR subsequently scans peptide–MHC I complexes outside of the PLC. However, because of the common binding mode, Tsn and TAPBPR might compete with each other for MHC I binding under certain conditions (79, 81). TAPBPR could also load epitopes derived from signal peptides onto MHC I, since it has a preference for HLA-A2 allomorphs, some of which are able to bind signal-sequence peptides (81, 86). Because TAPBPR operates outside of the PLC, it might act mainly as a chaperone recycling MHC I in regions where the concentration of optimal high-affinity peptides is lower than in the immediate vicinity of the TAP transporter (83). This theory seems to be supported by experimental evidence showing that TAPBPR increases the duration of MHC I–PLC interaction (81); this also points to a direct influence of TAPBPR activity on the PLC-mediated antigen presentation pathway. Furthermore, a more pronounced chaperone activity of TAPBPR is consistent with the finding that TAPBPR appears to bind substrate MHC I molecules with higher affinity than Tsn (79); however, the tighter binding could also be indicative of a more stringent peptide selection by TAPBPR.

Although the cellular process of catalyzed MHC I peptide loading turns out to be much more intricate than previously thought and the exact role of TAPBPR remains unknown, the line of evidence gained so far strongly suggests that TAPBPR represents a second peptide editor, in addition to Tsn. Due to the sequence and structural homology between TAPBPR and Tsn, the similar MHC I binding mode, and the shared biochemical characteristics, future studies of the interplay between TAPBPR and MHC I are expected to not only deepen our understanding of TAPBPR itself but also provide key information on the catalytic mechanism of Tsn.

## Tsn/TAPBPR and HLA-DM: Two Paths to a Common Goal

In the case of MHC class II, the non-polymorphic class II-like molecule HLA-DM is the chaperone that catalyzes peptide proofreading (87–91). It facilitates dissociation of class-II-associated invariant chain peptides (CLIP) in late endosomes, stabilizes empty MHC II, and catalyzes selection of high-affinity binders from a pool of endocytosed antigens. Through peptide proofreading, DM is able to promote presentation of peptides with half-lifes of more than 2 days (92). Remarkably, as has been gleaned from *in vitro* biochemical and crystallographic experiments with a mutated MHC II (93) and from an MHC II (DR)–DM complex structure, DM functions by interacting with the MHC II molecule close to the N-terminus of the peptide (94). HLA-DM induces a reorientation of a tryptophan in the P1 pocket of HLA-DR that normally interacts with the P1 anchor residue of the peptide. Additional structural changes in the vicinity of the P1 pocket stabilize the empty pocket: for example, a phenylalanine of HLA-DR moves into a position that is normally occupied by the P1 anchor residue of the peptide. Furthermore, an asparagine that stabilizes the P2 peptide backbone in peptide–DR complexes becomes engaged with a rearranged glutamate. As these regions of the MHC II are crucial for high-affinity peptide binding and the rearrangements render key residues inaccessible, this explains the facilitated peptide dissociation. At the same time, these rearrangements stabilize the empty part of the binding groove and thereby contribute to the chaperoning effect of DM. Only high-affinity peptides capable of competing with rearranged DR residues for access to the P1 pocket and the P2 site induce DM dissociation and get selected for presentation on the cell surface.

**Figure 5:**
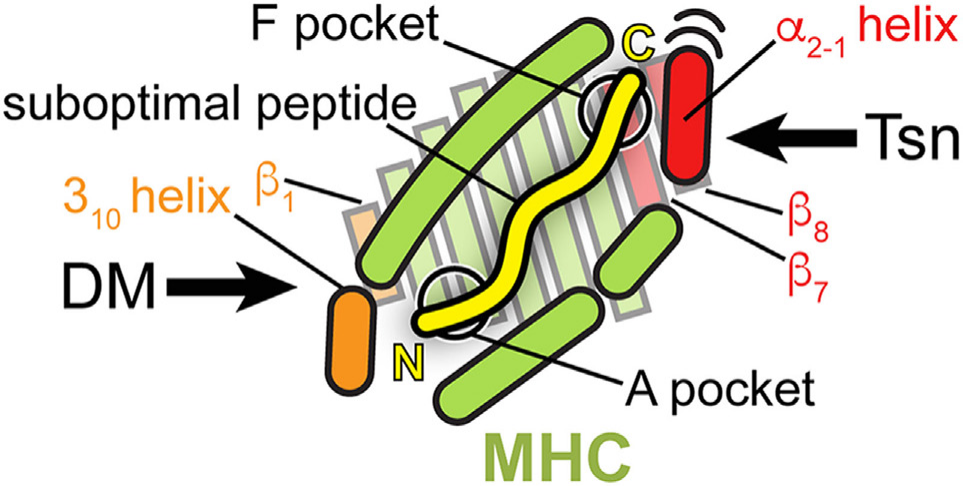
**Peptide proofreading by tapasin (Tsn) and DM—a structural comparison**. A top view of the MHC peptide-binding groove is shown schematically, highlighting structural elements, which the two peptide editors interact with during catalysis (orange: DM; red: Tsn). Bound peptide is indicated by a yellow line (N, N-terminus; C, C-terminus). Please note that the cartoon, including the length of the peptide, is a simplified depiction combining features of class I and class II MHC. Structural elements of MHC interacting with DM are close to the N-terminus of the peptide; those elements, which Tsn acts on, are in the vicinity of the F pocket of MHC I at the C-terminus of the peptide.

Thus, the proteins catalyzing peptide proofreading of class I and class II MHC molecules, Tsn, TAPBPR, and DM, share key features: (i) they preferentially bind and stabilize MHC molecules that are empty or loaded with low-affinity peptides, (ii) they accelerate peptide exchange, favoring high-affinity epitopes, (iii) and they achieve the chaperoning effect and selection of high-affinity peptides by directly interacting with structural elements flanking the peptide-binding groove. But while Tsn and TAPBPR presumably engage the MHC binding groove at the C-terminus of the peptide, DM acts at the N-terminus of the peptide (Figure 5) and has a high affinity for DR molecules loaded with peptides lacking N-terminal residues including the P1 anchor. Interestingly, a monoclonal antibody (mAb 64-3-7) has been described that is able to distinguish between peptide-deficient and peptide-loaded MHC I by recognizing a short epitope near the peptide N-terminus. The epitope is characterized by a tryptophan and a methionine that become solvent-exposed upon peptide dissociation. Thus, MHC I and MHC II appear to share some structural features in their peptide-receptive state at the binding region of the peptide N-terminus, e.g., a solvent-exposed tryptophan (71, 94). Nevertheless, Tsn and TAPBPR have adopted a different mode of interaction with the peptide-binding groove.

In essence, both types of exchange catalysts lower the energy barrier for peptide dissociation and stabilize the empty binding groove in a state which only high-affinity epitopes are able to go past. However, in the case of Tsn and TAPBPR, catalysis seems to involve a tug-of-war over the α_2-1_ helix of MHC I, whereas in the case of DM, exchange catalyst and peptide mainly compete for residues around the P1 pocket of the MHC II molecule (Figure 5).

## Summary and Future Directions

The recently obtained insights into peptide proofreading described in this review have significantly advanced our knowledge of this fundamental process in adaptive immunity and underline the importance of malleable structural elements and plasticity in the involved protein interaction partners. However, a comprehensive understanding of the molecular determinants in MHC I peptide editing will only be achieved once an experimentally determined structure of a Tsn–MHC I and/or TAPBPR–MHC I complex is available. Crystal structures of these two complexes will also help to fully comprehend the fact that different MHC I allomorphs exhibit varying degrees of Tsn/TAPBPR dependence and that Tsn and TAPBPR have different allomorph specificities. As has previously been noted (83), Tsn and TAPBPR might be decisive factors in shaping adaptive immune responses, since T cell receptors are able to recognize many different peptide–MHC I complexes. A thorough analysis of the mechanisms that underlie catalyzed peptide editing will therefore be critical to obtain a complete picture of immune recognition events in adaptive immunity governing such important processes as tumor surveillance, infection control, and transplant rejection. This might enable mechanism-based strategies to manipulate antigen presentation for therapeutic purposes, e.g., attenuating the processes in autoimmune diseases and upregulating antigen presentation in a targeted manner in cancer immunotherapy, antimicrobial therapies, and vaccination (95–97).

## Author Contributions

CT and RT wrote and edited the manuscript.

## Conflict of Interest Statement

The authors declare that the research was conducted in the absence of any commercial or financial relationships that could be construed as a potential conflict of interest.
